# Anti-MOG autoantibodies pathogenicity in children and macaques demyelinating diseases

**DOI:** 10.1186/s12974-019-1637-7

**Published:** 2019-11-30

**Authors:** Che Serguera, Lev Stimmer, Claire-Maelle Fovet, Philippe Horellou, Vanessa Contreras, Nicolas Tchitchek, Julie Massonneau, Carole Leroy, Audrey Perrin, Julien Flament, Philippe Hantraye, Joanna Demilly, Romain Marignier, Pascale Chrétien, Bert‘t Hart, Jean Boutonnat, Clovis Adam, Roger Le-Grand, Kumaran Deiva

**Affiliations:** 1 Commissariat à l’Energie Atomique (CEA), Institut de biologie François Jacob, Molecular Imaging Research Center (MIRCen), 92265 Fontenay-aux-Roses, France; 2 Institut national de la santé et de la recherche médicale (INSERM), MIRCen, UMS 27, 92265 Fontenay-aux-Roses, France; 3 Asfalia Biologics, Institut du Cerveau et de la Moelle épinière (ICM), Hôpital Pitié-Salpêtrière, Paris, France; 4 CEA, Inserm UMR 1184 and Institut de biologie François Jacob, Infectious Diseases Models for Innovative Therapies (IDMIT), Université Paris-Sud, 92265 Fontenay-aux-Roses, France; 5 Assistance Publique-Hôpitaux de Paris, Hôpital Bicêtre, Pediatric Neurology Department, National Referral Center for Rare Inflammatory Brain and Spinal Diseases, Hôpitaux Universitaires Paris-Sud, Paris, France; 6 Hôpital Neurologique Pierre Wertheimer, Service de Neurologie, Sclérose en plaques, pathologies de la myéline et neuro-inflammation, CHU de Lyon, 69677 Bron Cedex, France; 7 Immunology Department AP-HP, Hôpitaux Universitaires Paris-Sud, Le Kremlin Bicêtre, France; 8 Department of Immunobiology, Biomedical Primate Research Centre (BPRC), Rijswijk, The Netherlands; 9 Department Biomedial Sciences of Cells and Systems, University Medical Center Groningen, Groningen, The Netherlands; 10 CHU Grenoble-Alpes - TIMC UMR CNRS 5525, Grenoble, France; 11 Lab. de Neuropathologie, GHU Paris-Sud - Hopital Bicêtre, 94270 Le Kremlin Bicêtre, France

**Keywords:** Anti-MOG IgG, Cytokines, Complement, Demyelination, Brain inflammation, CSF

## Abstract

**Background:**

Autoantibodies against myelin oligodendrocyte glycoprotein (anti-MOG-Abs) occur in a majority of children with acquired demyelinating syndromes (ADS) and physiopathology is still under investigation. As cynomolgus macaques immunized with rhMOG, all develop an experimental autoimmune encephalomyelitis (EAE), we assessed relatedness between anti-MOG-Abs associated diseases in both species.

**Methods:**

The study includes 27 children followed for ADS and nine macaques with rhMOG-induced EAE. MRI lesions, cytokines in blood, and CSF at onset of ADS or EAE, as well as histopathological features of brain lesions were compared.

**Results:**

Twelve children with anti-MOG-Abs ADS (ADS MOG+) and nine macaques with EAE, presented increased IL-6 and G-CSF in the CSF, whereas no such signature was found in 15 ADS MOG−. Furthermore, IgG and C1q were associated to myelin and phagocytic cells in brains with EAE (*n* = 8) and in biopsies of ADS MOG+ (*n* = 2) but not ADS MOG− children (*n* = 1). Macaque brains also revealed prephagocytic lesions with IgG and C1q depositions but no leukocyte infiltration.

**Conclusions:**

Children with ADS MOG+ and macaques with EAE induced with rhMOG, present a similar cytokine signature in the CSF and a comparable aspect of brain lesions indicating analogous pathophysiological processes. In EAE, prephagocytic lesions points at IgG as an initial effector of myelin attack. These results support the pertinence of modeling ADS MOG+ in non-human primates to apprehend the natural development of anti-MOG-associated disease, find markers of evolution, and above all explore the efficacy of targeted therapies to test primate-restricted molecules.

## Introduction

More than 50% of acquired demyelinating syndromes (ADS) in children are associated to myelin oligodendrocyte glycoprotein antibodies (anti-MOG-Abs). Anti-MOG-Abs are frequent in optic neuritis (ON), transverse myelitis (TM), acute demyelinating encephalomyelitis (ADEM), or neuromyelitis optica spectrum disorder (NMOSD), but are rare in multiple sclerosis (MS) [1]. About 40% of ADS associated to anti-MOG-Abs (MOG+) evolve as a non-MS relapsing disease reluctant to conventional treatments, with cognitive disabilities in 20% of these children [2].

MOG is a CNS protein located at the outermost lamellae of myelin, and the extracellular domain of MOG or MOG peptides are efficiently used to induce brain restricted inflammatory demyelinating experimental autoimmune encephalomyelitis (EAE) in animals, the reference model of ADS [3].

Mouse EAE helps to understand the genetic and immune processes of autoimmunity [4], while non-human primates (NHP) models recapitulate the complex interplay between environment and the immune response. Moreover, macaques are phylogenetically closer to humans, which makes them uniquely suitable to test new therapies with antibodies or cytokines retaining functional and structural features restricted to primates.

Cynomolgus macaques immunized with recombinant human MOG (rhMOG) in incomplete Freund’s adjuvant (IFA) develop an acute encephalomyelitis, with brain magnetic resonance imaging (MRI) and demyelinating lesions reminiscent to that described in ADS [5].

To assess relatedness between anti-MOG-Abs-associated encephalomyelitis in macaques and children, we performed a comparative analysis between species with emphasis on cytokine production at disease onset and IgG and complement deposition in lesions. We report similar inflammatory processes in either species related to the presence of anti-MOG-Abs. This work contributes to our understanding of immunopathology of ADS associated with anti-MOG-Abs and substantiates the value of NHP for the setting of prospective therapies for ADS with anti-MOG-Abs (ADS MOG+).

## Materials and methods

### Study design

To assess the pathogenic role of anti-MOG-Abs in humans and macaques in the course of encephalomyelitis, we compared radiological, immune, and histologic parameters of nine animals with EAE and 27 humans with ADS of which 12 with anti-MOG-Abs. We used samples available in our respective collections consisting of three main groups of nine macaques with EAE, 15 children with ADS without anti-MOG-Abs, and 12 children with ADS with anti-MOG-Abs. As for comparisons between these groups, no previous data allowed to calculate sample size effect, we evaluated sample size through the resource equation method, which states that an acceptable degree of freedom (DF) for estimation of error with ANOVA ranges between 10 and 20. DF is calculated through the formula DF = (*n* (number of subjects) × *k* (number of groups)) − *k*. For each group, the DF was equal to 24 (EAE), 42 (ADS MOG+), and 33 (ADS MOG−), all above 20 indicating in each case an adequate size to assess statistical differences between groups [6].

### Patients and ethics

Twenty-seven children followed for a first episode of ADS in the national referral center for neuroinflammatory disease in children, Hôpitaux Universitaires Paris-Sud, Hôpital Bicêtre, from 2006/01/01 to 2014/31/12, and who had blood and/or CSF sample at onset, were included. An acute episode of demyelinating disease was defined as described [7]. Ten patients samples (five MS, three ADEM, two ON had been already involved in a previous study) [8]. Three patients, one from the 27 children group and two new patients lacking blood and CSF samples, were added to this study for histological analysis as they had a brain biopsy following an atypical pseudo-tumoral presentation. Patient 1 was a 7-year-old girl at the time of the biopsy (3 months after ON at onset, when she relapsed as severe ataxia with a pseudo-tumoral right peduncular lesion) and was later diagnosed as relapsing ADS with anti-MOG-Abs (RADS MOG+). Patient 2, a 2-year-old boy, presented an acute hemiplegia and alteration of consciousness with a pseudo-tumoral lesion on the right hemisphere. He had a brain biopsy at onset and was later diagnosed with monophasic ADEM with anti-MOG-Abs (ADS MOG+). Patient 3, a 4-year-old boy, presented an ADEM at onset which relapsed 1 month later. The third attack (gait difficulties) occurred 9 months later without encephalopathy and MRI evolved as an extensive leukodystrophy and a brain biopsy was performed at month 19. Extensive metabolic and genetic workup remained negative; there were no criteria for pediatric onset MS and the patient was diagnosed as non-MOG relapsing ADS (RADS MOG−). The brain biopsy was performed in this child at distance from steroids (> 30 days of steroids); he had no other immunomodulatory treatment in between). All human studies have been reviewed by the appropriate ethics committee and have therefore been performed in accordance with the ethical standards laid down in an appropriate version of the 1964 Declaration of Helsinki. The national cohort of first demyelinating episode “Kidbiosep 2004” (N° 910506) was authorized by the Commission National de l’Information et des Libertés and an informed consent form was obtained for all included children.

### Animals and ethics

Samples from the MIRCen collection including plasma, CSF, and brain tissue as well as MRI images and clinical data from nine adult cynomolgus macaques (*Macaca fascicularis*) immunized with rhMOG/IFA were included in this study. Immunization protocols, clinical observations, CSF, and blood collection methods were described previously [9]. Seven to 9-year-old cynomolgus macaques were purpose-bred animals imported from a licensed primate-breeding center in Mauritius (Cynologics Ltd, Port Louis, Mauritius). Following European directive 2010/63/UE and French regulations, the project was performed in an agreed user establishment (agreement number C 92-032-02), with an institutional permission obtained from the French Ministry of Agriculture after evaluation by an ethical committee (*2015081710528804vl*). All procedures were performed in compliance with CEA’s animal welfare structure under veterinary care at all times. Macaques were observed double-blinded daily from immunization until the end of the experiment; they were scored daily for EAE outcome directly by the experimenter or through webcam surveillance, using a semi-quantitative functional scale grading disease severity form grade 0 (healthy), grade 0.5 (loss of appetite, vomiting), grade 1 (apathy), grade 2 (ataxia, sensory loss and/or visual problems), grade 2.5 (hemiparesis or paraparesis), grade 3 (hemiplegia or paraplegia), grade 4 (quadriplegia), to grade 5 (protraction), all signs corresponding to classical neurological nosology conducing to ADS diagnosis (Additional file 1: Table S1 and S2). Ethical endpoints of EAE are based on evaluation of disease gravity, with increased severity shortening time of the experiment ultimately ending with animal euthanasia [9]. One animal (EAE_c, Additional file 1: Table S2) was treated with 140 mg/kg per day of corticoids (Solumedrol ®) intravenous for 3 days starting at the very onset of disease, grade 0.5 with tremor of lower limbs confirmed with MRI.

### Treatment of cerebrospinal fluid and plasma

In children, CSF samples were obtained by lumbar puncture before treatment at onset of encephalomyelitis and processed by the laboratory hospital. For our study, we used 0.5 ml of CSF, centrifuged and stored at − 80 °C. Plasma was collected from 10 ml of blood in heparinized tubes and stored at − 80 °C.

In macaques, CSF was sampled before immunization, as well as at EAE onset, for up to 500 μl per puncture. A 150 μl aliquot was used for analysis and the remaining was centrifuged and stored at − 80 °C free of cells. Venous blood samples were collected every week, starting before the first immunization with rhMOG/IFA and until the end of experimentation, as well as at EAE onset and before euthanasia, with a maximum volume of up to 15% of the total blood volume per animal per month. Blood was centrifuged and plasma was stored at − 80 °C.

### Immunological analysis

#### Measurement of anti-MOG-Abs levels

Plasma was tested by cell-based assay (CBA) for titration of antibodies to cell surface MOG epitopes as described [8]. Briefly, 1.5 × 10^5^ HEK293A cells transfected with the pIRES2-DsRed2-human MOG were incubated with patient or macaque plasma at dilutions ranging from 1:10 to 1:160 for 1 h at 4 °C. Cells were incubated with FITC conjugated anti-IgG H + L Fab'2 secondary antibody (Kallestad FITC conjugate, Bio-Rad, Marnes la Coquette, France) for 15 min at 4 °C and fixed. A total of 5 × 10^4^ events per sample were recorded on a FACS Canto II instrument and analyzed with Flow Jo software (Ashland, OR, USA). Binding was expressed as mean fluorescence intensity (MFI). Levels of specific antibody binding in transfected cells were expressed as ΔMFI determined by the subtraction of MFI obtained with HEK293A control cells from that obtained with HEK293MOG+ cells. When a positive signal was found with transfected cells incubated with patients plasma diluted at 1:160, the sample was considered MOG-Abs positive (MOG+) [8].

#### Cerebrospinal fluid analysis

For children, CSF aliquots were sent for routine CSF analysis: cell count, oligoclonal band, glucose, lactate, total protein, and viral diagnosis. For macaques, the presence of cells in the CSF was evaluated and quantified in 100 μl after Cytospin™ 4 Cytocentrifuge (ThermoFisher Scientific, Villebon sur Yvette, France) centrifugation. Slides were stained with routine May-Grünwald Giemsa (MGG) special stain. Overall cell density and cell type were recorded.

#### Cytokines assessments

Fifteen cytokines, for which kit cross-reaction with macaques’ epitopes had been previously assessed at IDMIT, were measured in plasma and CSF with multiplex technology, MILLIPLEX MAP human Cytokine Magnetic Bead Panel–Customized Premixed 13 Plex (Bulk) Packaging (IL-1β, GM-CSF, G-CSF, IL-2, IL-4, IL-6, IL-8, IL-10, IL-12p40, IL-17A, TNFα, IFNγ), and MILLIPLEX MAP TGFß Magnetic Bead 3 Plex Kit–Immunology Multiplex Assay (TGFβ 1, TGFβ 2, and TGFβ 3), as two separate dosages, following supplier (Merckmillipore, Burlington, MA, USA) guidelines using a Bioplex 200 (BioRad, Hercules, CA, USA). The quantification of all samples was performed all together at the end of study samplings.

### MRI evaluation

In children, brain MRI at onset was done during the first week and reviewed by a trained pediatric neurologist and a radiologist. The other items added were as follows: (1) for distribution, multiple lobe lesions: lesions present in more than one lobe (frontal, temporal, parietal, and occipital); bilateral lesion: lesions in both hemispheres; symmetric lesions: symmetric distribution of lesions; (2) signal intensity was evaluated in Spin Echo T2-weighted images, fluid-attenuated inversion recovery (FLAIR) Spin Echo, or T1-weighted images sequences; (3) for appearance of the lesion, fuzzy lesions: lesions lacking clear limits/borders. Similar sequences and images were compared to MRI from cynomolgus macaques at EAE onset for comparative analysis.

For macaques, magnetic resonance imaging (MRI) acquisitions were performed on a horizontal 7T Agilent scanner (Palo Alto, CA, USA), using a surface coil for transmission and reception (RAPID Biomedical GmbH, Rimpar, Germany). T2-weighted images were acquired using a high-resolution 2D fast spin-echo sequence. Lesion segmentation was performed on the T2-weighted images. During scanning, animals were anesthetized and maintained in a dedicated stereotaxic frame. The respiratory rate was monitored (SA Instruments Inc., Stony Brook, NY, USA) and body temperature was maintained at 37 °C using heated waterbed.

### Histology

#### Tissue preparation

Human brain biopsies and 4% PFA-perfused macaque brains were processed to paraffin blocks, cut and stained with hematoxylin eosin stain (HE). Brain lesions were scored according to their overall severity, number, size, morphological characteristics, as well as according to the intralesional myelin loss.

#### Immunohistochemistry

Immunohistochemistry was used to investigate intracerebral IgG and IgM distribution. Briefly, paraffin wax-embedded tissues from humans or macaques were dewaxed in xylene and hydrated through graded alcohols. Endogenous peroxidase activity was suppressed by 3% H_2_O_2_ in PBS. Subsequently, sections were incubated with goat anti-IgG (anti-human IgG (gamma chain), 1/100, SAB3701291, Sigma-Aldrich, Saint-Louis, MI, USA), with rabbit anti-IgM (anti-human IgM, mu chain, 1/250, A0425), with polyclonal rabbit anti-Human C1q (1/100, A0136, Dako, Les Ulis, France), with anti-MBP (1/100, SMI94R BioLegend, San Diego, CA, USA), with rabbit anti-IBA1 (1/100, ab178846, Abcam, Cambridge, UK), or with mouse anti-CD68 (1/150, ab 955, KP1, Abcam). Tissues were then incubated with secondary antibodies coupled to fluorochromes Alexa 488 or Alexa 594 (ThermoFisher, Villebon-sur-Yvette, France). Alternatively, biotinylated rabbit-anti-goat, rabbit-anti-mouse, or goat-anti-rabbit antibodies were used as secondary antibodies, for 30 min at room temperature, followed by the avidin-biotin-peroxidase complex (Vectastain Elite ABC Kit, Vector Laboratories, PK 6100; Burlingame, CA, USA). Positive antigen-antibody reactions were visualized by incubation with 3,3-diaminobenzidine-tetrahydrochloride (DAB)–H_2_O_2_ in 0.1 M imidazole, pH 7.1 for 5 min, followed by slight counterstaining with hematoxylin.

### Statistical analysis

Statistical analyses and graphical representations were obtained using Prism 5 (GraphPad Software, Inc). Student’s *t* test was used to compare two groups of values. The two-sided one-way ANOVA test with Tukey’s multiple comparison test was used to compare three groups or more values. Heatmaps were generated using R software (R Foundation for Statistical Computing, Vienna, Austria). A chi-squared test was performed to compare frequencies of lesions detected with MRI per brain regions. Hierarchical clustering represented by dendrograms were generated based on the Euclidian distance and using the complete linkage method.

### Data availability statement

All data files enclosing values or images corresponding to clinical characteristic of patients or monkeys including routine biological measurements, MRI, as well as dosages of anti-MOG-Abs and cytokines are available upon request. Tissue sections from patient or animal lesions and samples of plasma or CSF can be shared upon request depending on availability and purpose.

## Results

### Diseases characteristics in humans and macaques

In this study, with the purpose to compare the characteristics of encephalomyelitis among two species of primates, we analyzed nine macaques with EAE together with 27 patients with ADS. All macaques immunized with rhMOG/IFA declared EAE between 11 and 211 days post immunization (dpi) and disease manifested through signs of neurological dysfunction mimicking major clinical and radiological features of human ADS (Additional file 1: Table S1,), of variable severity that was diagnosed and graded at each round of observation [9] (Additional file 1: Table S2).

At disease onset, among the 27 patients, ten were diagnosed as MS, seven as ADEM, one as NMOSD, and nine as CIS (six ON, two TM, and one hemiplegia). At last follow-up, ten were diagnosed as MS, five as ADEM, six as CIS (three ON, two TM, one hemiplegia), two as NMOSD, and four as non-MS relapsing demyelinating diseases with anti-MOG-Abs all named (ADS MOG+) (Additional file 1: Table S3). Among the 27 patients, 15 had ADS without anti-MOG-Abs (MOG-) and 12 had ADS with this biomarker (MOG+). Most were girls (85%); none of them had anti- AQP4 IgG. Among the 12 ADS MOG+, six had a monophasic course (ADEM = 1, TM = 2, ON = 3) and six a relapsing course (RADS MOG+ = 6) (Table 1).

**Table 1:** Children involved in this study. Characteristics of included children at last follow-up (FU)

Demographic	ADS *n* = 27	MS *n* = 10	ADEM *n* = 5	ADS MOG+ *n* = 6	Others *n* = 6
Sex M/F	6: 21	1: 9	1: 4	2: 4	2: 4
Age years, mean, SD	11.1 ± 4.1	12.8 ± 1.9	7.9 ± 5.5	8.9 ± 4.3	13.1 ± 2.6
EDSS mean, SD	0.7 ± 1.2	1.4 ± 1.6	0 ± 0	0.7 ± 0.8	0 ± 0
CSF					
Cell > 10 mm^3^	11	3	2	5	1
Prot > 0.5 g/dL	7	3	1	2	1
OCB (*n* = 22)	8	6/10	1/3	1/5	0/4
FU years, mean, SD	3.0 ± 1.6	3.5 ± 1.2	3.2 ± 0.9	3.8 ± 2.3	1.3 ± 0.7
Anti-AQP4	0	0	0	0	0
Anti-MOG	12	0	1	6	5

*ADS* acquired demyelinating syndrome, *MS* multiple sclerosis, *ADEM* acute demyelinating encephalomyelitis, *ADS* MOG+ non-MS relapsing demyelinating diseases with anti-MOG-Abs, *Others*:optic neuritis (*n* = 3), transverse myelitis (*n* = 2), hemiplegia (*n* = 1). Expanded Disability Status Scale (EDSS), oligo-clonal bands (OCB), follow-up (FU)

**Fig. 1:**
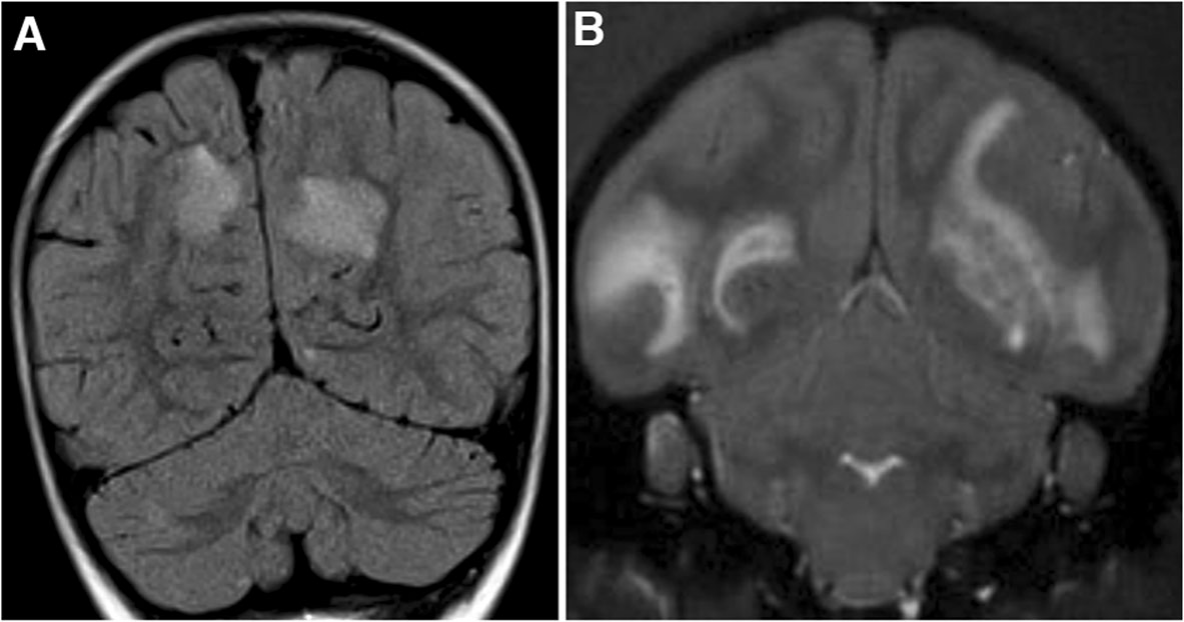
Brain MRI in human MOG+ ADS and macaque EAE at onset of disease. **a** Coronal section of a FLAIR acquisition of brain MRI showing a bilateral hyperintense signal in both hemispheres, principally spread in the cortical white matter of a child with ADEM. **b** Coronal section of a T2* acquisition of brain MRI showing a bilateral hyperintense signal in both hemispheres, principally spread in the cortical white matter of a macaque with EAE. Note in both cases the similar location, extension and aspect of lesions with poorly defined borders.

At disease onset, MRI was abnormal in all children and animals. When comparing MRI of patients, we observed that distribution of lesions in patients with ADS MOG+ seemed to be globally different from that of patients with ADS MOG−: there were significantly less periventricular lesions, or perpendicular to the great axis of the corpus callosum, or juxta cortical, or cortical as well as less gadolinium enhancing lesions, or focal well defined, infra tentorial and brain stem lesions (Additional file 1: Table S4.1). Comparing the localization and aspect of brain lesions between patients with ADS MOG+ and EAE only revealed a higher lesion load in macaques as compared to humans (Additional file 1: Table S4.2). Lesions in EAE displayed a rather diffuse aspect with poorly defined borders as often observed in ADEM (Fig. 1). Thus, features of brain lesions at MRI appeared to be rather different between patients with ADS MOG− and ADS MOG+ but rather similar between lesions from patients with ADS MOG+ and that from macaques with EAE.

### Anti-MOG IgG levels in humans and macaques

**Fig. 2:**
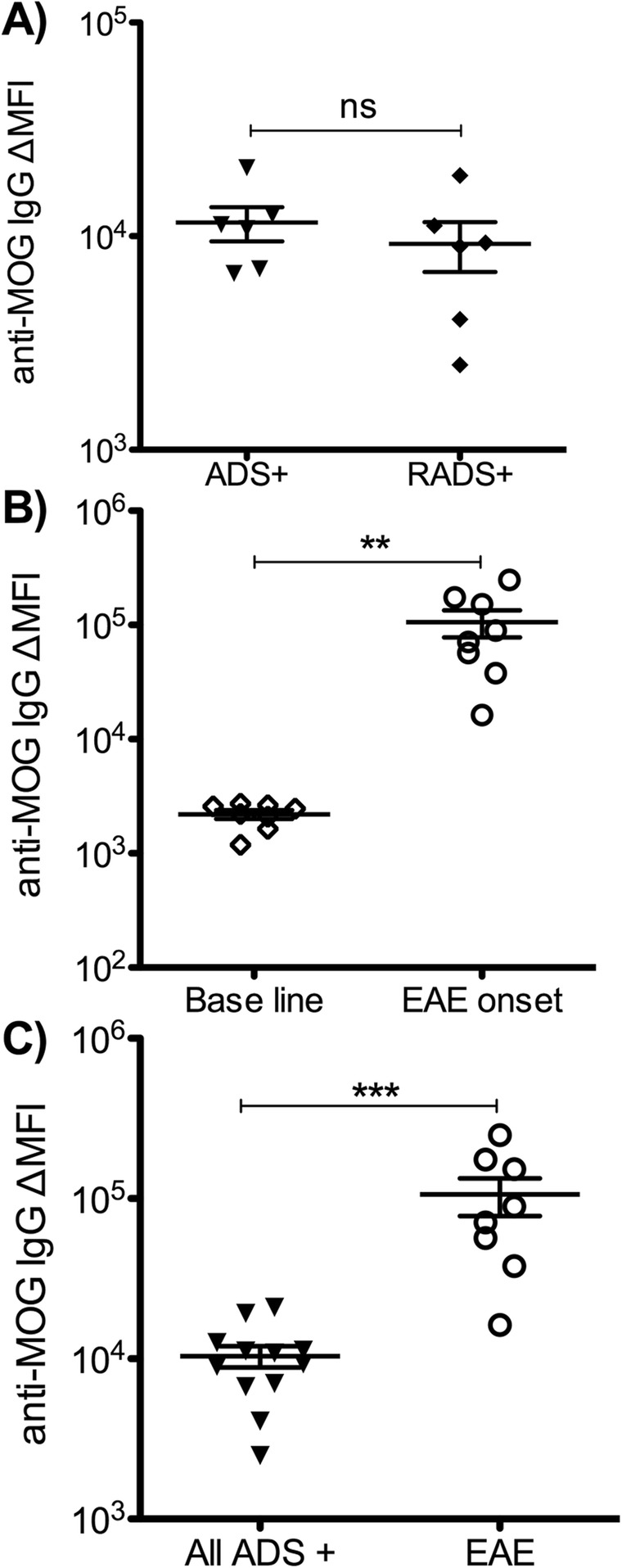
Levels of anti-MOG IgG in human ADS and macaque EAE. Dosage of anti-MOG IgG measured with cell-based assay (CBA). **a** Delta mean fluorescence intensity (ΔMFI) of anti-MOG IgG in children with ADS with anti-MOG IgG either monophasic (ADS+) or relapsing (RADS+). **b** Levels of anti-MOG IgG in macaques before immunization (baseline) and at onset of EAE. **c** Comparison of levels of anti-MOG IgG between children with ADS/RADS MOG+ (pool of monophasic and relapsing ADS MOG+), and EAE at onset of diseases. Statistics, unpaired *t* test two-tailed. *P* value summary, *p ≤ 0.05; **p ≤ 0.01; ***p ≤ 0.001

Using a cell-based assay (CBA), we compared the levels of anti-MOG IgG between ADS MOG+, RADS MOG+, and macaque EAE. We observed no difference in levels of anti-MOG IgG between patients with ADS MOG+ and RADS MOG+ (*p* = 0.47) (Fig. 2a). In all animals immunized with rhMOG/IFA, we measured an important increment of anti-MOG IgG between baseline and EAE onset (*p* = 0.0023) (Fig. 2b). Moreover, macaques immunized with rhMOG/IFA produced about 12 times more anti-MOG IgG than patients with ADS/RADS MOG+ (*p* = 0.0004) (Fig. 2c). The presence of higher levels of anti-MOG IgG in the monkey model than in patients could explain why EAE is a more severe disease than that observed in ADS/RADS MOG+.

### Cytokine assessments in macaques and humans with EAE and ADS

**Fig. 3:**
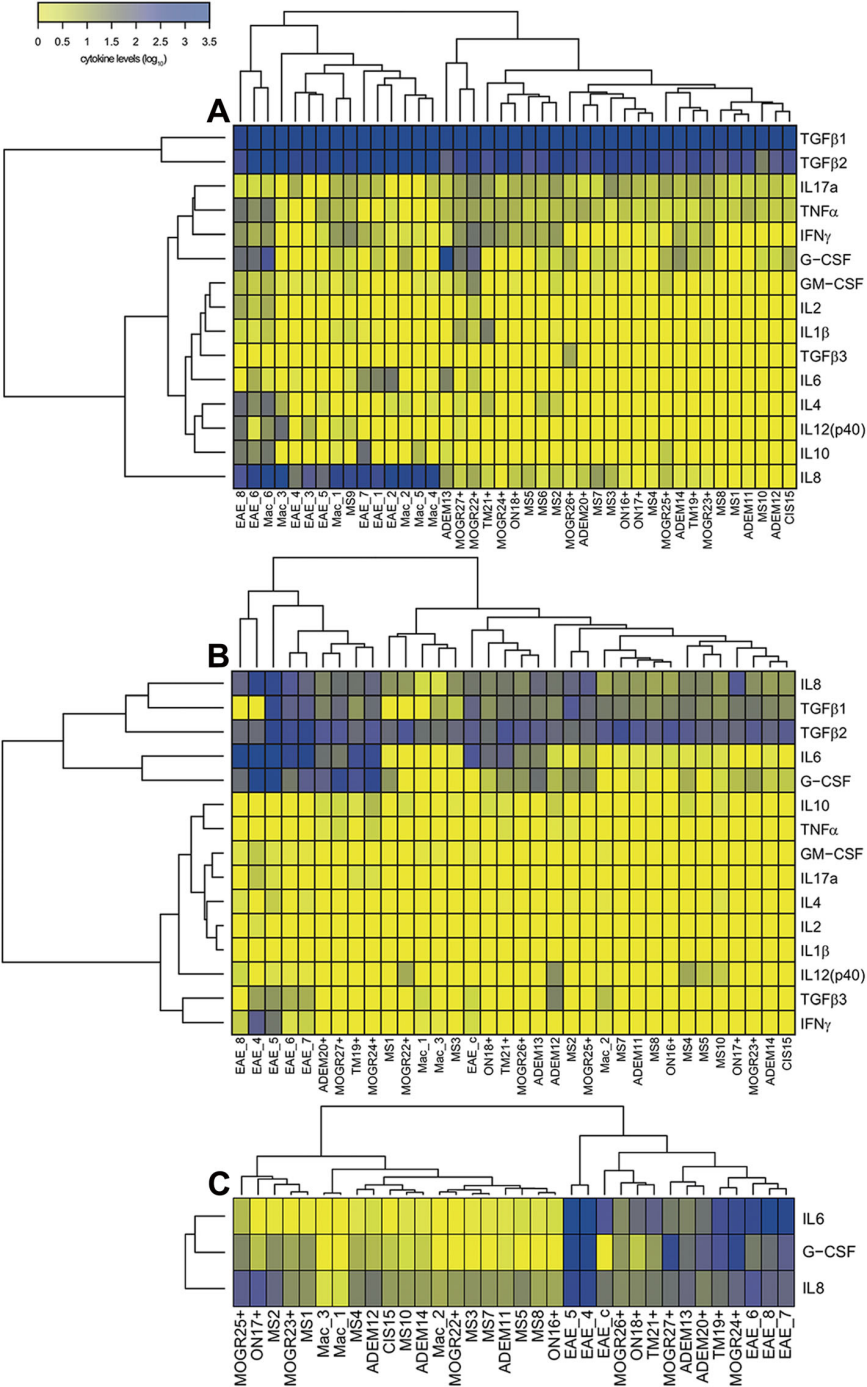
Heatmap representation of human and macaque cytokine levels at disease onset. The heatmap representing expression levels of 15 cytokines measured in plasma and the CSF of patients and animals at onset of ADS or EAE. Hierarchical clustering of cytokine levels, represented by dendrograms, were performed to gather samples having similar cytokine profiles and cytokines having similar expression patterns in the dataset. **a** In plasma, the hierarchical clustering defines two groups of individuals. Note that group 1 (left) gathers all samples from macaques either at the baseline or at disease onset and 1 child with MS (MS9). Group 2 (right) aggregates all other 26 humans included in this study. **b** In the CSF, the hierarchical clustering also defined two main groups of individuals. Group A (left) gathers five macaques at EAE onset and four children at onset of ADS MOG+; group B (right) contains all animals at base line and one EAE treated with corticoids at onset of disease, but also all patients with ADS MOG− and seven patients with ADS MOG+ (the three ON, the two NMOSD, one ADEM, one TM, and one RADS MOG+) who had elevated IL6 or G-CSF or both but in a lower magnitude as compared to patients from group A. Note that one subgroup includes the one macaque with EAE treated with corticoids and three patients with ADS MOG+. **c** In the CSF dosage, the hierarchical clustering restricted to the three cytokines IL6, G-CSF, and IL8 shows two main groups, group C1 (left) with all patients ADS MOG− but one, four patients with ADS MOG+ (two NMOSD (MOGR), two ON and one ADEM), and all animals at baseline; group C2 (right) includes all animals at EAE onset, seven patients with ADS MOG+ and one patient with ADS MOG− (ADEM). Each individual is represented by a number and the name of its disease. Experimental autoimmune encephalomyelitis (EAE), acute demyelinating encephalomyelitis (ADEM), neuromyelitis optica spectrum disorder (NMOSD), clinically isolated syndrome (CIS), multiple sclerosis (MS), optic neuritis (ON), idiopathic transverse myelitis (TM), or MOG relapsing disease (MOGR) for atypical ADS MOG+. EAE_c refers to the CSF collected 11 days after EAE onset and treatment with 140 mg/kg of corticoids per day during the first 3 days after disease declared. (Mac) refers to healthy macaques at baseline. The sign (+) indicates patients with anti-MOG IgG

We then asked if macaques with EAE and children with ADS MOG+ would produce the same type of cytokines detected in adults with anti-MOG associated diseases [10]. For that, we dosed a panel of 15 cytokines in the plasma and the CSF of monkeys with EAE and patients with ADS, using antibodies with cross recognition of the cytokines in both species. Moreover, the panel of cytokines was meant to infer the implication of subsets of T lymphocytes with a defined phenotype Th1: TNFα and IFNγ; Th2: IL-2 and IL-4; Th17: IL-6, IL-8, IL-17A, GM-CSF, and G-CSF; Tregs: IL-10 and TGFβ1, or other proinflammatory cytokines: IL-1β and IL-12p40. To assess if children with ADS MOG+ and monkeys with EAE would co-segregated in common groups defined by variation of the same cytokines, we ordered the values of these dosages through hierarchical clustering. Cytokine levels in plasma split samples in two exclusive groups with humans on one side and macaques on the other. This indicates that plasma levels of cytokines rather defined species but not disease. Indeed, each species mainly differed in its respective plasma content of IL-8 and IL-10 that were 50 and 12 times more elevated in macaques, either healthy or ill, and G-CSF that was about 20 times higher in humans than in macaques (Fig. 3a). By contrast, the same analysis of cytokine levels in the CSF revealed mixed groups including children and macaques. The main left group included five macaques at onset of EAE and four patients with ADS MOG+, while the main right group was composed of samples from 21 patients and four macaques (three healthy animals and one with EAE but treated with corticoids that notably segregated with the three patients ON18+ (ADS MOG+), TM21+ (ADS MOG+), and MOGR 22+ (RADS MOG+)). Individuals in the left groups had higher levels of IL-8, G-CSF, and IL-6 than those in the right group. By contrast, patients with ADS MOG+ in the right group had lower levels of IL-6 or G-CSF than those in the left group (Fig. 3b). Simpler hierarchical clustering including only the three cytokines IL-6, IL-8, and G-CSF further evidenced the relatedness between EAE and ADS MOG+, as all animals with EAE segregated with seven patients with ADS MOG+ but only one ADS MOG−. This establishes that EAE and ADS MOG+ present a similar pattern of cytokine elevation that is different from ADS MOG− (Fig. 3c).

**Fig. 4.**
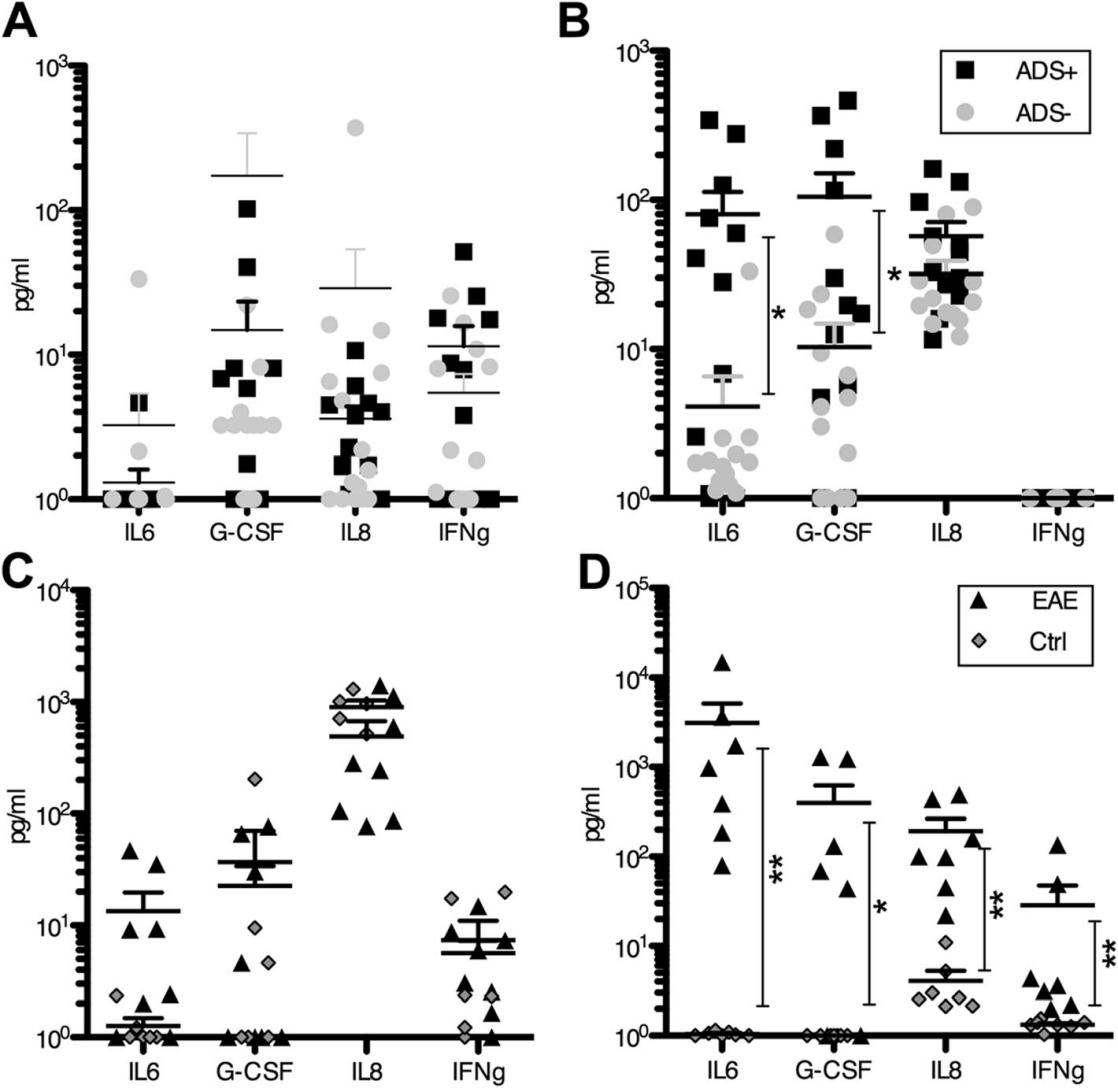
Most expressed cytokines at disease onset in humans and macaques. Cytokines IL6, G-CSF, IL8, and IFNγ were found mostly elevated in the plasma and the CSF of human ADS and macaque EAE. **a** No significant differences are observed in levels of the four cytokines in plasma of patients with ADS MOG− (gray circles, ADS−) or ADS MOG+ (black squares, ADS+) at onset of disease. **b** In the CSF of patients with ADS MOG+, IL6 and G-CSF are significantly increased as compared to that in patients with ADS MOG−. **c** In macaque plasma at EAE onset, cytokine levels are not significantly different than that measured before immunization. **d** In the CSF of macaques at onset of EAE, IL6, G-CSF, IL8, and IFNγ were found significantly increased as compared to the baseline. Statistics, Mann-Whitney test two tailed. *p < 0.05; **p < 0.01.

We next assessed if patients with ADS MOG−, ADS MOG+, and macaques with EAE expressed different amounts of the cytokine G-CSF, IL-6, IL-8, and IFNγ in the plasma and the CSF. In patients’ plasma or their CSF, none of these cytokines appeared as a marker of monophasic or relapsing disease. We thus compared the plasma levels of cytokines of the 12 patients with ADS or RADS MOG+ to that of the 15 patients with ADS MOG−, and observed no significant difference between the two groups (Fig. 4a). By contrast, in the CSF of patients with ADS/RADS MOG+, we observed significantly increased IL-6 (*p* = 0.015) and G-CSF (*p* = 0.018) but not IL-8 (*p* = 0.086) as compared to those measured in the CSF of patients with ADS MOG- (Fig. 4b). In macaque plasma, none of these four cytokines were found elevated at EAE onset as compared to the baseline (Fig. 4c). On the contrary in the CSF, IL-6 (*p* = 0.002), G-CSF (*p* = 0.011), IL-8 (*p* = 0.002), and IFNγ (*p* = 0.002) were all found increased at onset of EAE as compared to the levels measured at baseline (Fig. 4d). Thus, a characteristic elevation of IL6 and G-CSF in the CSF of ADS MOG+ and EAE points at a similar pathophysiological process of disease in both conditions rather involving a proinflammatory process driven by Th17 lymphocytes as observed in adults with anti-MOG-associated disease [10]. In addition, higher levels of IL-6 (48×), G-CSF (5.3×), and IL-8 (4.6×) in the CSF of macaques at onset of EAE as compared to that measured in the CSF of children with ADS/RADS MOG+ points at a more severe inflammatory response in monkeys. This is possibly associated with increased numbers of encephalitogenic lymphocytes producing these cytokines and higher levels of anti-MOG IgG.

### Histopathology of lesions in EAE and ADS

Depending on diseases, the anatomical sites of lesions and their respective dissemination within the CNS varies [11], which usually determines the nature and magnitude of the neurological deficits. Main aspects of active lesions of cynomolgus macaques are well characterized [5]. However, although the main histopathological hallmark is similar in EAE and ADS, and consists in perivenular inflammation and demyelination, the precise content in immune effectors varies among diseases (Additional file 1: Table S1).

**Fig. 5.**
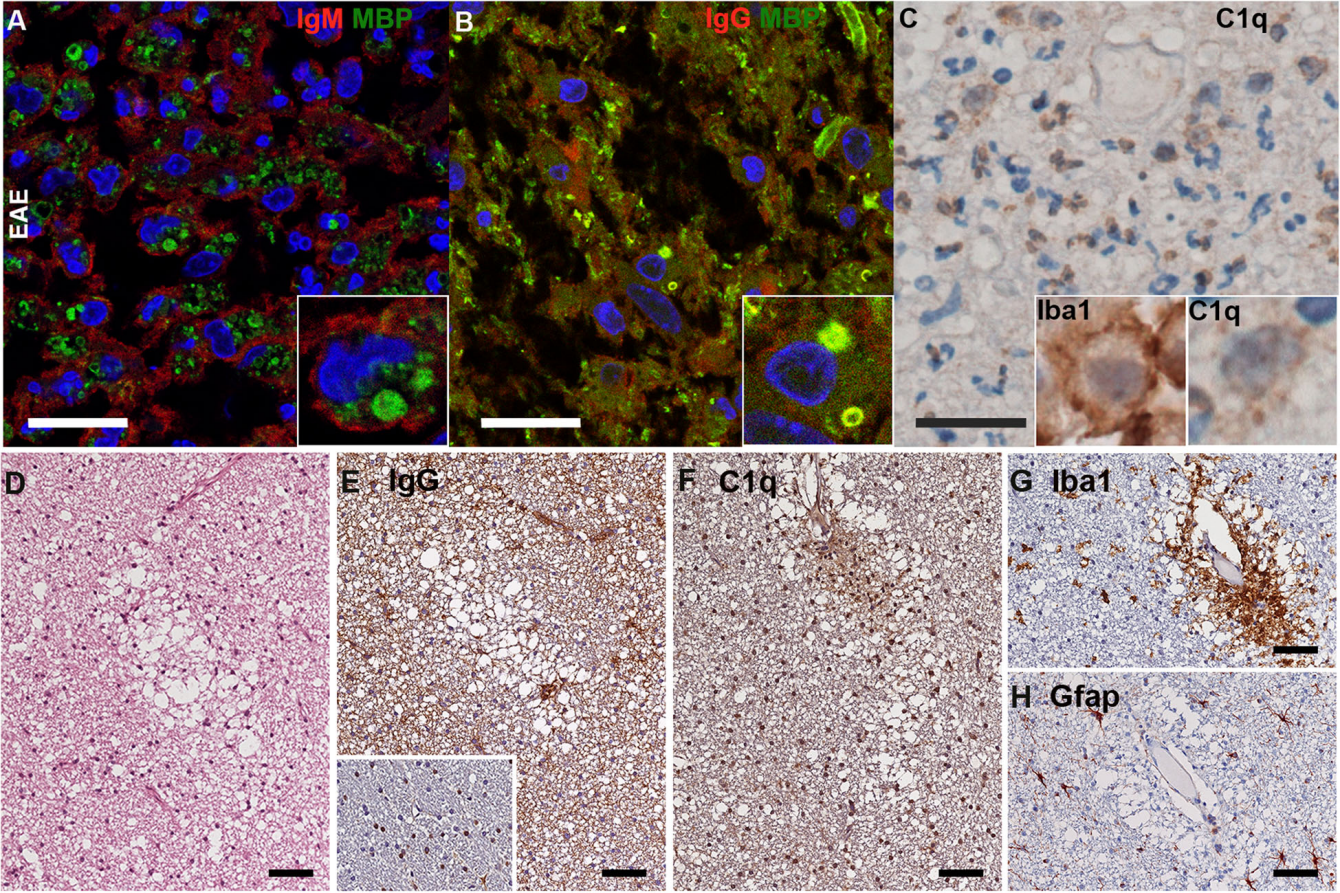
Immunohistology of brain lesions of macaque EAE. **a**–**c** Tissue sections from brain lesions were stained to detect IgG, IgM, and the myelin protein MBP (confocal microscopy) or the marker of activated microglia/macrophages IBA1 or the complement factor C1q (bright field microscopy). (scale bars 20 μm). **a** Immunofluorescence (IF) staining of MBP in green and IgM in red shows the non-overlapping presence of MBP and IgM; inset: a higher magnification shows a microglia/macrophage coated with IgM and containing MBP. **b** IF of MBP in green and IgG in red, shows colocalized MBP and IgG; inset: a higher magnification shows a microglia/macrophage with MBP-positive myelin particles colocalized with IgG. **c** Intralesional presence of C1q (brown precipitates) within neutrophils and microglia/macrophages; left inset: a higher magnification of an activated microglia/macrophage labeled with IBA1, right inset: a higher magnification of a macrophage with intracytoplasmic labeling of C1q. **d**–**h** Prephagocytic lesions in macaque EAE detected as myelin vacuolization in a lesion proximal to an active demyelinating lesion in the white matter in the subcortical area of a macaque brain (bright field microscopy, scale bars 50 μm). **d** HE stains shows evenly distributed brain cells in the white matter and myelin vacuolization and the absence of leukocyte infiltrate. **e** Diffuse IgG deposits surrounding the white matter. Inset shows that the myelin in immediate proximity of this prephagocytic lesion is free of IgG labeling. **f** Diffuse C1q deposition on vacuolized myelin. **g** The same area shows the presence of widespread and perivascular IBA1+ activated microglia. **h** In these lesions, normal GFAP expression indicates absence of astrogliosis

Here, we explored if immunoglobulins IgM and IgG were present within inflammatory lesions of macaques with EAE or that of children with ADS MOG+ or ADS MOG−, and if they were coupled to myelin and complement. In EAE, lesions contained numerous activated macrophages/microglia and polymorphonuclear cells containing phagocytosed MBP+ myelin. Intralesional staining of IgM were present at the membrane of macrophages/microglia, but did not colocalize with MBP, indicating that IgM in macaques EAE do not recognize native myelin (Fig. 5a). In contrast, IgG were present within active lesions colocalizing with MBP on myelin sheets and inside macrophages/microglia (Fig. 5b). The complement protein C1q showed a pattern of distribution similar to that of IgG in active lesions, covering myelin fibers and within activated macrophages/microglia, expressing IBA1 (Fig. 5c). Depositions of IgG and C1q were detected in all lesions from macaque EAE where they were assessed (seven out of nine for IgG and five out nine for C1q) (Additional file 1: Table S2). These results indicate that macaques developing EAE produce IgG able to recognize and opsonize brain myelin and favor its phagocytosis.

In three macaque brains, prephagocytic lesions could be observed at relative proximity from active lesions, similar to that described in type II MS brain[12]. These were characterized by myelin vacuolization but without leukocyte infiltrate (Fig. 5d). They contained diffuse IgG and C1q deposits surrounding white matter (Fig. 5e, f). These “early” lesions also contained IBA1+ activated microglia uniformly distributed in tissue or gathered at perivascular location (Fig. 5g), but a complete absence of astrogliosis (Fig. 5h), while GFAP expression is always observed in active lesions of macaques [5]. This points at an early role of IgG and complement in triggering inflammation and myelin destruction in macaque EAE.

**Fig. 6.**
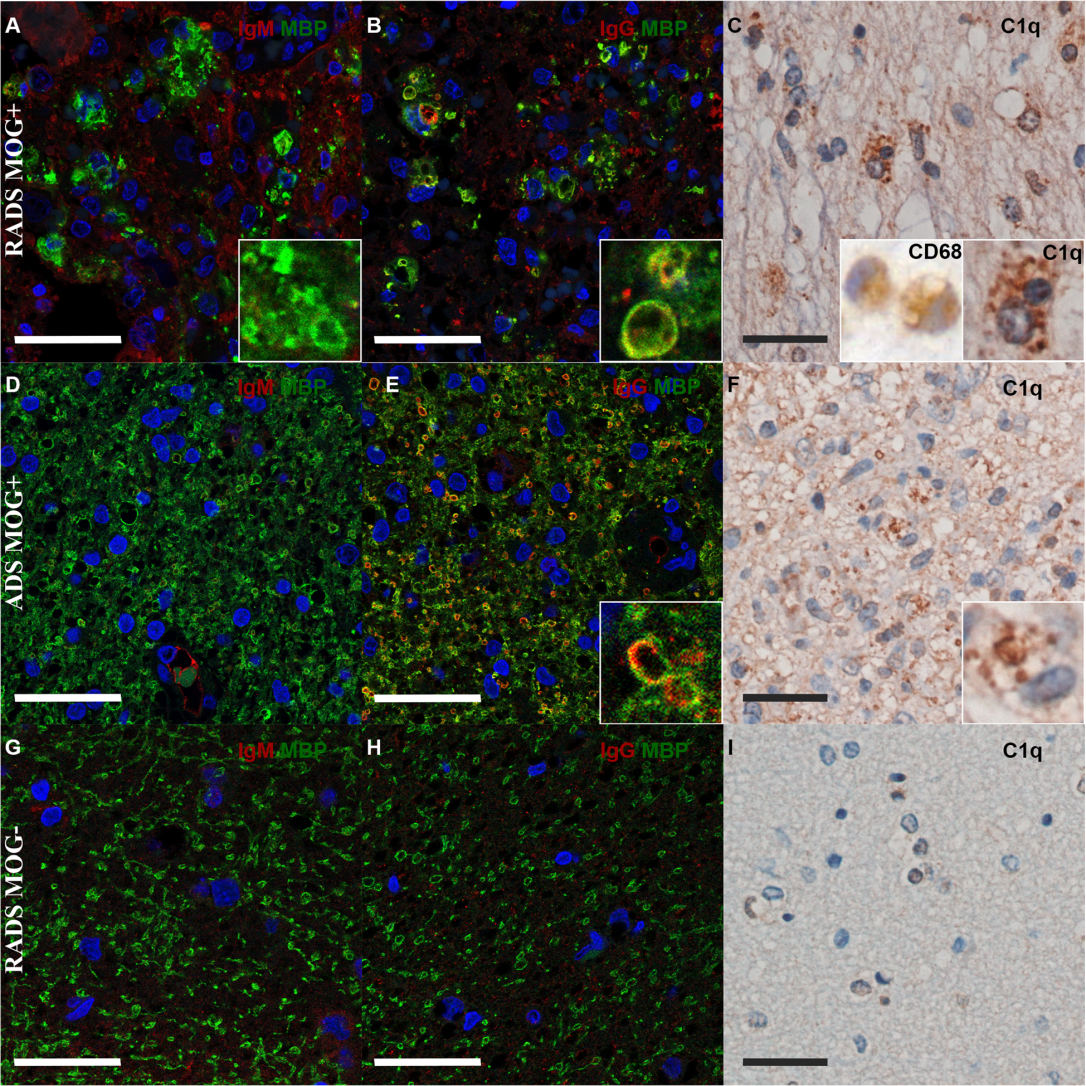
Immunohistology of brain lesions of children ADS. Tissues corresponding to brain lesions of three patients with ADS/RADS, either MOG+ (*n* = 2) or MOG− (*n* = 1). One lesion from a patient with RADS MOG+ (**b, c**), another lesion from a patient with a monophasic ADEM (ADS MOG+) (**d**–**f**), or a RADS MOG− (**g**–**i**), were stained to detect IgG, IgM, and the myelin protein MBP (confocal microscopy) or the activated microglia/macrophage marker CD68, or the complement factor C1q (bright field microscopy) (scale bars 20 μm.) **a** In the tissue section from patient 1, immunofluorescence (IF) staining of MBP in green and IgM in red shows the non-overlapping presence of MBP and IgM in the parenchyma; inset: a higher magnification shows a microglia/macrophage containing MBP. **b** IF of MBP in green and IgG in red shows co-localization of both signals in the parenchyma; inset: a higher magnification shows a microglia/macrophage with inclusions stained with MBP and IgG. **c** C1q staining in brown appears intracellular within microglia/macrophages; left inset: a higher magnification of an activated microglia/macrophage labeled with CD68, right inset: a higher magnification shows a microglia/macrophage with intracytoplasmic labeling of C1q. **d** IF of MBP in green and IgM in red shows conserved architecture of tissue and no or scarce IgM. **e** MBP in green and IgG in red show overlapping signals in the parenchyma of patient 2; inset: a higher magnification shows a microglia/macrophage with inclusions of mixed MBP and IgG. **f** C1q staining in brown within brain lesions appears intracellular; in the bottom right corner, a higher magnification shows a microglia/macrophage with intracytoplasmic labeling of C1q. **g** IF of MBP in green reveals myelin vacuolization and IgM in red shows scarce presence of IgM in the tissue and coating membranes of phagocytic cells. **h** MBP in green and IgG in red show scarce amount of IgG in tissue and absence of colocalization of MBP and IgG. **i** No C1q labeling is appreciable within this lesion. In all IF, nuclei are stained in blue with DAPI

To assess if lesions of children with ADS MOG+ would also contain IgG and complement, we performed a histopathological analysis of biopsies of three children, two with ADS MOG+ (one RADS and one monophasic) and one with RADS MOG−. The lesion of the first patient with a RADS MOG+ (MOGR27+) contained IgM in the extracellular space of an unstructured white matter with scarce MBP left except within macrophages/microglia (Fig. 6a). IgG, instead, were seen within macrophages/microglia together with MBP (Fig. 6b), as well as the complement protein C1q (Fig. 6c). In these lesions, activated macrophages/microglia were identified morphologically and through labeling of the myeloid marker CD68 (Fig. 6c). In the lesion of a second patient with a monophasic ADEM MOG+, no IgM were detected, while MBP staining revealed a rather well-conserved tissue architecture (Fig. 6d). In this lesion, IgG were present coating myelin fibers and within macrophages associated to MBP (Fig. 6e). In this patient also, C1q had a pattern of distribution similar to that of IgG, at the periphery of myelin fibers and within macrophages (Fig. 6f). In clear contrast from the two lesions from patients with RADS/ADS MOG+, the biopsy of a lesion from a child with RADS MOG− showed scarce deposition of IgM or IgG and absence of C1q amidst a mildly unstructured tissue with macrophages infiltrate (Fig. 6g–i).

These results establish that IgG and complement are associated to myelin in brain lesions of macaque EAE and in lesions of patients with ADS MOG+, which further point at a direct correlation between the presence of circulating anti-MOG IgG and a role for these IgG as a mediator of brain inflammation in both species. In addition, in a biopsy from a patient with ADS MOG−, the absence of IgG and complement depositions illustrate such an inflammatory lesion exempt of antibodies and complement, which possibly characterize part of all ADS MOG−.

## Discussion

We report that cynomolgus macaques immunized with rhMOG/IFA develop an acute encephalomyelitis with a characteristic neuro-inflammatory profile similar to that observed in children with ADS MOG+, but which is different from that seen in children with ADS MOG− including all cases of MS. This is evidenced through a matching cytokine profile in the CSF at onset of EAE and ADS MOG+. In addition, we also show analogous histologic depositions of IgG and complement in demyelinated brain lesions of EAE and ADS MOG+. This points to a similar pathogenic role of anti-MOG-IgG in macaque EAE and in children ADS MOG+ that trigger a complement-mediated immune response against myelin. The present comparison also establishes the value of this macaque model for translational studies related to anti-MOG associated disease.

### Cytokines signature in the CSF of ADS MOG+ and EAE

Previous studies of pediatric and adult ADS indicate that MOG+ diseases rather respond to immunosuppression, B cell depletion, or intravenous IgG but not to MS-modifying treatments, putting humoral immunity at the center of the inflammatory response [2, 13]. In fact, during the acute phases of ADS MOG+ and ADS AQP4+ NMOSD, cytokines related to B-lymphocytes, Th17 CD4+ cells, and neutrophils have been recurrently found elevated in the CSF [8, 10, 13–15], while MS patients rather express Th1 cytokines and chemokines [13]. In the present study, it is notorious that the cytokines IL6 and G-CSF are found increased in the CSF of children with ADS MOG+ and in EAE but not in ADS MOG−. This highlights the predominant role of Th17 lymphocytes triggering demyelination through humoral and innate immunity in children, as observed in adults with or anti-MOG IgG-associated disease [8, 10, 13] and in NMOSD with anti-AQP4 IgG [13–15]. The absence of cytokines elevation in blood at onset of EAE or ADS indicates that the inflammatory response is restricted to the CNS, once antibodies and leukocytes have crossed the blood-brain barrier.

### Demyelinating lesions in children with ADS MOG+ and macaques with EAE

Histopathology analysis of brain biopsies of seven adults with ADS MOG+ have been previously reported, showing the concomitance of a perivascular inflammatory infiltrate with IgG and complement depositions on myelin sheets and within macrophages characterizing demyelinating plaques with preserved axons and tissue structure [16–21]. Echoing these evidences, we show that IgG and complement are found coating myelinated axons or within macrophages in inflammatory lesions of two children with ADS or RADS MOG+. These lesions exhibit disparate severity with tissue structure found conserved in the monophasic form but destructed in the relapsing MOG+ diseases. This was correlated to respective transient or sustained levels of circulating anti-MOG IgG, suggesting a causal link between continuous antibodies production and relapses [22]. It is noteworthy that macaque EAE is globally more severe than ADS MOG+ as it evolves to a lethal outcome more often than to a resolving or a relapsing disease [9]. We speculate that such severe encephalomyelitis in EAE is conceivably associated to higher levels of circulating anti-MOG-Abs than that dosed in patients, explaining the more pronounced infiltration of lesions by phagocytic cells and the increased release of cytokines and chemokines, as compared to ADS MOG+. Such a strong response in the macaque model is conceivably due to immunization with massive amounts of antigen, efficiently breaking a precarious peripheral tolerance to MOG and inducing a more uniform and wider expansion of MOG-reactive CD4+ T cells and their priming into Th1 or Th17 phenotype than it may happen in post-infectious or otherwise idiopathic ADS MOG+. In fact, immunosuppression attenuates EAE in non-human primates, favoring milder forms of disease clinically more alike remitting-relapsing ADS [23].

### Prephagocytic lesions

In macaques, we observed prephagocytic lesions in the white matter characterized by vacuolized myelin associated to IgG and C1q and a complete absence of leukocyte infiltrate. These images are reminiscent of tissue changes described in the periphery of active MS plaques and they are interpreted as a nascent type II demyelinating lesions in MS [12] and EAE in marmoset [24]. This observation suggests that the first myelin disturbance is induced by antibody opsonization and complement activation prior to the recruitment of myelomonocytic cells and gives further consistency to the view of anti-MOG IgG as being a central pathogenic element in ADS MOG+. The presence of prephagocytic lesions indicates that very few autoreactive T cells are sufficient to disrupt the blood brain barrier and permit subsequent antibody-mediated inflammatory events as demonstrated with combination of adoptive transfer of patients anti-MOG antibodies and autoreactive T cells in mouse models of EAE [4].

### Unresolved questions

The main limitation of this study concerns the small size of each group, especially the subgroups of ADS MOG+, or particular clinical entities as ADEM, NMOSD, ON, TM, or CIS. Thus, nonetheless, we provide convergent observations with other studies, concerning main inflammatory processes in the brain in the presence of anti-MOG-Abs [8, 10, 13, 15–17, 20, 25], the small size of each subgroup precluded further comparisons between specific diseases. Another weakness of our work is the absence of dosage of additional cytokines or chemokines of potential interest for the classification of diseases as those more specifically related to B cells recruitment and differentiation. Clearly, larger cohorts and high-throughput measurements are required to assess in each case the causes for relapses and to identify markers specific to each disease.

## Conclusions

ADS is a heterogeneous group of diseases presenting different clinical courses. In concert with previous observations made in adult patients, we describe a different cytokine profile in the CSF of children with ADS MOG+ compared to ADS MOG−. Local anti-MOG IgG deposits and complement activation in the perivenular white matter seem to play a central role in the pathogenesis of EAE and ADS MOG+, initiating and amplifying demyelination. Most importantly, the macaque EAE model recapitulates immune processes identified in ADS MOG+, including CSF cytokine signature and similar lesion appearance, establishing the medical value of such NHP model of ADS to study pathophysiological aspects of anti-MOG IgG-associated diseases. In addition, this monkey model is especially valuable to address the effectiveness of novel therapies, which due to phylogenetic distance cannot be completed in rodents [26].

## Supplementary information

**Additional file 1: Table S1.** Multiparameter comparison of EAE in macaques and human ADS. **Table S2.** Macaques involved in this study. **Table S3.** Disease evolution of children involved in this study. **Table S4.** Sites of lesions in patients MOG- vs. patients MOG+. Sites of lesions in patients MOG+ vs. macaques EAE.

## Data Availability

All data available upon request to authors. Materials can be shared depending on availability and purpose.

## Abbreviations

Abs: Antibodies
ADEM: Acute demyelinating encephalomyelitis
ADS MOG+: ADS with anti-MOG-Abs
ADS: Acquired demyelinating syndromes
EAE: Experimental autoimmune encephalomyelitis
IFA: Incomplete Freund’s adjuvant
MOG: Myelin oligodendrocyte glycoprotein
MRI: Magnetic resonance imaging
MS: Multiple sclerosis
NMOSD: Neuromyelitis optica spectrum disorder
ON: Optic neuritis
RADS MOG+: Relapsing ADS with anti-MOG-Abs
RADS: Relapsing ADS
rhMOG: Recombinant human MOG
TM: Transverse myelitis
