# Supplementary material for: Anti-MOG autoantibodies pathogenicity in children and macaques demyelinating diseases
Source: J Neuroinflammation. 2019 Nov 30;16:244. doi: 10.1186/s12974-019-1637-7 (PMC6884758; doi:10.1186/s12974-019-1637-7)
Supplement: Supplementary file 1 — Additional file 1: Table S1. Multiparameter comparison of EAE in macaques and human ADS. Table S2. Macaques involved in this study. Table S3. Disease evolution of children involved in this study. Table S4. Sites of lesions in patients MOG- vs. patients MOG+. Sites of lesions in patients MOG+ vs. macaques EAE. [file 12974_2019_1637_MOESM1_ESM.doc]

**Supplementary tables**

|  | **Present model of EAE** | **ADEM** | **NMOSD** | **CIS** | **MS** | **ON**  [**http://eyewiki.aao.org/**](http://eyewiki.aao.org/) | **ITM** |
| --- | --- | --- | --- | --- | --- | --- | --- |
| **Clinical appearance** | Acute encephalomyelitis (*1*) | Acute encephalomyelitis with encephalopathy (*2*) | Acute optic neuritis and-or myelitis (*3*) | First episode of acute encephalomyelitis | Chronic encephalomyelitis | Acute unilateral optic neuritis | Acute myelitis |
| **MRI lesion profile (T2-hyperintense lesions)** | Blurred, large bilateral lesions in WM and deep GM (*4*) | Blurred, large bilateral lesions in WM and deep GM (*2*) | >1/2 optic nerve or optic chiasma; intramedullary over 3 vertebrae (*3*) | Variable site, usually monofocal (*5*) | Variable site, dissemination in space & time (*6*) | Enhancement of the orbital optic nerve | Central T2 hyperintense SC lesion extending over more than 2/3 of cord section (*7*) |
| **Distribution** | Brain multifocal, bilateral, non-symmetric (*1*) | Brain multifocal, bilateral, non-symmetric (*2, 8*) | Optic nerve bilateral, spinal cord longitudinal (*3*) | ON, SC, BS, Cm (*5*) | ⦥1periventricular, cortical or juxtacortical, or infratentorial; ⦥2 SC (*6*) | Optic nerve | Over more than 2/3 of the cross-sectional area of the cord (*7*) |
| **LCR abnormalities** | Mild polymorph- or mononuclear pleocytosis and fewer lymphocytes (*1*) | Lymphocytes and mild mono- and polymorphonuclear pleocytosis (*2, 8*) | Mild polymorph- or mononuclear pleocytosis, fewer lymphocytes (*3, 9*) | Low to moderate pleocytosis, lymphocytes and macrophages (*5, 10*) | Low to moderate pleocytosis, lymphocytes and macrophages. (*6*) | Not affected unless atypical (MS, NMOSD, infectious) | Low to moderate pleocytosis as MS or NMOSD (*11*) |
| **Oligoclonal bands** | N.A. | <30% (*2, 8*) | <20% (*3, 9*) | >70% (*12*) | >80% | 15-80% (*13*) | Rare & transient unless MS-related (*11*) |
| **MOG / AQP4 IgG** | Anti-MOG IgG: All (this article,*4*) | Anti-MOG IgG <30% (*14, 15*) | 20% anti-MOG / 70% anti-AQP4 (*3, 9*) | Described although as rare as in MS (*16*) | Rare (*14, 17, 18*) | 10-20% in relapsing ON (*19*) | 6%/20% in LETM 10%/80% in NMO (*20*) |
| **Treatment**  **(**[**http://**](http://nationalmssociety.org/)  [**nationalmssociety**](http://nationalmssociety.org/)  [**.org**](http://nationalmssociety.org/)**)** | N. A. | 1) Corticosteroids 2) Plasmapheresis 3) IV immune globulin | 1) Corticosteroids 2) Plasmapheresis 3) Immunomodulators 4) Rituximab 5) Azathioprine | 1) No treatment 2) Corticosteroids | 1) Corticosteroids 2) IFN-beta 3) Monoclonal Abs 4) Immunomodulators 5) Symptomatic | 1) No treatment 2) IV corticosteroids | 1) Corticosteroids 2) Plasmapheresis 3) IV immune globulin 4) Chemotherapy |
| **Relapses or progression** | 15-20% (*1*) | 20-50% (*21, 22*) | 60-90% (*3, 9*) | 10-85% (*5*) | 100% | 20%-60% | 30%-60% (*23, 24*) |
| **Inflammation** | Severe, necrotizing polymorph- or mononuclear (*1*) | Mild to severe mono- and polymorphonuclear cells (*25-28*) | Severe, necrotizing mononuclear & neutrophils & eosinophils (*29*) | Mild to moderate lymphocytic and mononuclear (*10, 16*) | Mild to moderate lymphocytic and mononuclear (*30, 31*) | Mild to moderate lymphocytic (*32*) | Mild to moderate mononuclear and lymphocytic (*11*) |
| **Demyelination / axonal damage** | WM perivenular / often damaged by necrosis (*1*) | WM perivenular / relatively preserved (*25-28*) | ON, myelin (WM & GM) / often damaged by necrosis (*29*) | Perivenular (WM & GM) / damaged (*10, 16*) | Perivenular / variable (*30, 31*) | Perivenular / preserved | Central cord & symmetric/some axonal loss (*11*) |
| **IgG and complement deposits** | IgG associated to myelin (this article) | IgG & Cp deposit in a patient’s anti-MOG+ (*25, 26*) | Important IgG & Cp deposit (*29*) | Occasional (*16*) | MS type II in ⦥ 50% (*30, 31*) | As in MS | Described in number of cases (*11*) |

**Supplementary table 1**. **Multiparameter comparison of EAE in macaques and human ADS.** Abbreviations: Experimental Autoimmune Encephalitis (EAE), Acute Demyelinating Encephalomyelitis (ADEM), Neuromyelitis optica Spectrum Disorder (NMOSD), Clinically Isolated Syndrome (CIS), Multiple Sclerosis (MS), Optic Neuritis (ON), Idiopathic Transverse Myelitis (ITM), White matter (WM), Grey matter (GM), Spinal Cord (SC), Longitudinally Extensive Transverse Myelitis (LETM), Complement (Cp).

| **Animal** | **Age (years)** | **Sex** | **EAE onset (dpi)** | **Clinical signs** | **CBA (ΔMFI)** | **IgG/C1q in lesions** | **Euthanasia dpi/grade (G)** |
| --- | --- | --- | --- | --- | --- | --- | --- |
| **EAE_1** | 7.9 | M | 11 | Paresis, ataxia | 15121 | ++/+ | 15/G4 |
| **EAE_2** | 8.6 | F | 24 | Coma | 35429 | +++/NA | 25/G5 |
| **EAE_3** | 7.8 | M | 30 | Paresis, ataxia, | 54086 | +++/NA | 34/G4 |
| **EAE_4** | 7.7 | F | 67 | Paresis, nystagmus | 68731 | ++/+++ | 223/G5 |
| **EAE_5** | 7.7 | F | 25 | Ataxia, paraplegia | 87488 | NA/+ | 27/G4 |
| **EAE_6** | 8.1 | F | 41 | Paresis, ataxia | 150623 | +++/NA | 43/G2,5 |
| **EAE_7** | 7.5 | M | 211 | Paresis, diarrhea | 172768 | +/+++ | 215/G2 |
| **EAE_8** | 8.3 | M | 66 | Paresis, ataxia | 245929 | +++/++ | 68/G4 |
| **EAE_9 (EAE_c)** | 5.7 | F | 30 | Paresis bilateral, optic neuritis | 12470 | NA/NA | 41/G3 |

**Supplementary table 2. Macaques involved in this study.** Named by numbers from 1 to 9. EAE_9 (EAE_c) is the animal treated with corticoids. Different features of EAE_1, 3, 4, 5, 6 and 7 have been described in (4), while EAE, 2, 8 and 9 are described for the first time. Age of animals at onset of disease. Sex of animals, male (M) and female (F). Day of onset of EAE ranges from 11 to 211 days post immunization (dpi). Clinical signs detected at onset of EAE. Values of ΔMFI of anti-MOG IgG measured at euthanasia. Appreciation of the relative amount of IgG and complement C1q present in inflammatory brain lesion as low (+), mild (++), high (+++) or not available (NA). Time of euthanasia in dpi/grade of disease severity (G) as graded from 0 to 5 as in (4). 0= no clinical signs; 0.5= loss of appetite, vomiting; 1= substantial reduction of general condition; 2 = ataxia, sensory loss and/or visual problems; 2.5= incomplete paralysis of one (hemiparesis) or two sides (paraparesis); 3= complete paralysis of one (hemiplegia) of two sides (paraplegia); 4= complete paralysis (quadriplegia); 5= moribund.

| **ADS** | **Diagnosis at onset** | **Diagnosis at the end of FU** | **# MOG-/+** | **Age (years)** | **Sex** |
| --- | --- | --- | --- | --- | --- |
| **MS MOG- (n=10)** | MS | MS | 1 | 10.9 | F |
|  | MS | MS | 2 | 12.6 | F |
|  | MS | MS | 3 | 14.6 | F |
|  | MS | MS | 4 | 15.0 | F |
|  | MS | MS | 5 | 12.4 | F |
|  | MS | MS | 6 | 14.0 | F |
|  | MS | MS | 7 | 13.3 | F |
|  | MS | MS | 8 | 14.6 | F |
|  | MS | MS | 9 | 8.9 | F |
|  | ON | MS | 10 | 12.2 | M |
| **Monophasic ADS MOG- (n=5)** | ADEM | ADEM | 11 | 2.3 | F |
|  | ADEM | ADEM | 12 | 14.1 | F |
|  | ADEM | ADEM | 13 | 11.8 | F |
|  | ADEM | ADEM | 14 | 9.1 | F |
|  | CIS | CIS | 15 | 15.5 | F |
| **Monophasic ADS MOG+ (n=6)** | ON | ON | 16+ | 12.5 | F |
|  | ON | ON | 17+ | 12.3 | F |
|  | ON | ON | 18+ | 7.6 | F |
|  | TM | TM | 19+ | 15.0 | F |
|  | ADEM | ADEM | 20+ | 2.0 | F |
|  | TM | TM | 21+ | 15.9 | F |
| **Relapsing ADS MOG+ (n=6)** | MS | RADS MOG+ | 22+ | 11.9 | F |
|  | ADEM | RADS MOG+ (NMOSD) | 23+ | 13.57 | F |
|  | ADEM | RADS MOG+ | 24+ | 3.2 | F |
|  | NMOSD | RADS MOG+ (NMOSD) | 25+ | 13.2 | F |
|  | ON | RADS MOG+ | 26+ | 5.3 | M |
|  | ON | RADS MOG+ | 27+ * | 6.8 | M |

**Supplementary table 3. Disease evolution of children involved in this study.** Patient groups of Acquired Demyelinating Syndromes (ADS) either monophasic or relapsing. Disease at onset or at last follow-up (FU) named by the acronym of their respective condition, Multiple Sclerosis (MS), Acute Demyelinating Encephalomyelitis (ADEM), Clinically Isolated Syndrome (CIS), Optic Neuritis (ON), Idiopathic Transverse Myelitis (TM), Neuromyelitis Optica Spectrum Disorder (NMOSD) or relapsing ADS MOG+ (RADS MOG+) for atypical IDD with anti-MOG IgG ; here NMOSD and atypical IDD MOG+ are all called MOGR. Patients are numbered from 1 to 27; seropositivity for anti-MOG IgG is indicated with (+). Age of children at onset of disease. Sex of patients, male (M) and female (F). (*) Patient included in histological analysis.

| Children | **ADS MOG-**  **n=15 (%)** | **ADS MOG+**  **n=12 (%)** | **P value** |
| --- | --- | --- | --- |
| **Periventricular lesion** | 12 (80) | 5 (42) | 0.04* |
| **corpus callosum** | 6(40) | 2 (18) | NS |
| **Perpendicular to corpus callosum** | 11(64) | 3(25) | 0.04* |
| **juxta-cortical lesion** | 11 (73) | 3(25) | 0.01* |
| **cortical** | 10(67) | 0 (0) | 0.01* |
| **deep gray matter** | 6(40) | 1 (8) | NS |
| **Gad enhancing** | 10 (66) | 2 (17) | 0.01* |
| **ring enhancement** | 6 (40) | 0 (0) | 0.02* |
| **hypoT1** | 10 (67) | 5 (42) | NS |
| **Lesion load (>75%)** | 2 (13) | 0 (0) | NS |
| **Focal** | 13 (87) | 5 (42) | 0.06 |
| **large** | 4 (27) | 3 (25) | NS |
| **well defined** | 13 (87) | 6 (50) | 0.02* |
| **pseudotumoral** | 0 (0) | 1 (8) | NS |
| **infra-tentoriel** | 12(80) | 4(33) | 0.01* |
| **Spinal lesion** | 7 (47) | 4 (33) | NS |
| **Multiple lobes** | 14(93) | 5(42) | 0.02* |
| **Brain stem lesion** | 9 (60) | 2(17) | 0.02* |

**Supplementary table 4.1.** **Sites of lesions in patients MOG- vs. patients MOG+.** Sites of hyperintense lesions detected with brain and spine MRI in all patients with ADS without anti-MOG IgG (ADS MOG-) or with anti-MOG IgG (ADS MOG+) involved in this study. Statistical evaluation of frequencies of lesions per area was performed using a chi-squared test.

|  | **Primates**  **n=8 (%)** | **Human**  **n=12(%)** | **P value** |
| --- | --- | --- | --- |
| **Periventricular lesion** | 3 (38) | 5 (42) | NS |
| **corpus callosum** | 0(0) | 2 (18) | NS |
| **Perpendicular to corpus callosum** | 0(0) | 3(25) | NS |
| **juxta-cortical lesion** | 6 (75) | 3(25) | NS |
| **cortical** | 3(38) | 0 (0) | NS (0.058) |
| **deep gray matter** | 0(0) | 1 (8) | NS |
| **Gad enhancing** | 1 (13) | 2 (17) | NS |
| **ring enhancement** | 0 (0) | 0 (0) |  |
| **hypoT1** | 5 (63) | 5(42) | NS |
| **Lesion load (>75%)** | 4(50) | 0(0) | 0.014* |
| **Focal** | 5 (63) | 5 (42) | NS |
| **large** | 7 (88) | 3 (25) | 0.02* |
| **well defined** | 2 (25) | 6 (50) | 0.0001*** |
| **pseudotumoral** | 1 (13) | 1 (8) | NS |
| **infra-tentoriel** | 5(63) | 4(33) | NS |
| **Spinal lesion** | ND | 5 (36) | NS |
| **Brain stem** | 4(50) | 2(17) | NS |

**Supplementary table 4.2.** **Sites of lesions in patients MOG+ vs. macaques EAE.** Sites of hyperintense lesions detected with brain and spine MRI in all macaques at EAE and in patients with ADS with anti-MOG IgG (ADS MOG+) involved in this study. Statistical evaluation of frequencies of lesions per area was performed using a chi-squared test.

**References**

| 1. L. Stimmer, *et al.*, *Vet Pathol* **55**, 27 (Jan, 2018).  2. M. Tardieu, Y. Mikaeloff, *Eur J Paediatr Neurol* **8**, 239 (2004).  3. J. de Seze, *et al.*, *Rev Neurol (Paris)* **172**, 256 (Apr-May, 2016).  4. K. G. Haanstra *et al.*, *J Neuroimmune Pharmacol* **8**, 1251 (Dec, 2013).  5. D. H. Miller, *et al.*, *Lancet Neurol* **11**, 157 (Feb, 2012).  6. A. J. Thompson *et al.*, *Lancet Neurol* **17**, 162 (Feb, 2018).  7. C. Goh, *et al.*, *J Magn Reson Imaging* **40**, 1267 (Dec, 2014).  8. D. L. Koelman *et al.*, *Neurology* **86**, 2085 (May 31, 2016).  9. D. M. Wingerchuk *et al.*, *Neurology* **85**, 177 (Jul 14, 2015).  10. C. Krishnan, A. I. *et al.*, *Front Biosci* **9**, 1483 (May 1, 2004).  11. A. Awad, O. Stuve, *Curr Neuropharmacol* **9**, 417 (Sep, 2011).  12. J. Kuhle *et al.*, *Mult Scler* **21**, 1013 (Jul, 2015).  13. N. Heussinger *et al.*, *Ann Neurol* **77**, 1076 (Jun, 2015).  14. A. Cobo-Calvo *et al.*, *J Neurol* **264**, 1945 (Sep, 2017).  15. A. K. Probstel *et al.*, *Neurology* **77**, 580 (Aug 9, 2011).  16. S. Jarius *et al.*, *Mult Scler* **22**, 1541 (Oct, 2016). | 17. A. M. Hacohen Y, *et al.*, *Neurol Neuroimmunol Neuroinflamm*, **2**, 81 (Apr, 2015).  18. S. Jarius *et al.*, *J Neuroinflammation* **15**, 88 (Mar 19, 2018).  19. J. Jitprapaikulsan *et al.*, *Ophthalmology*, (Apr 28, 2018).  20. R. Hoftberger *et al.*, *Mult Scler* **21**, 866 (Jun, 2015).  21. D. L. Koelman *et al.*, *Eur J Neurol* **24**, 391 (Feb, 2017).  22. Y. Mikaeloff *et al.*, *Eur J Paediatr Neurol* **11**, 90 (Mar, 2007).  23. R. C. Dale, F. Brilot, B. Banwell, *Curr Opin Neurol* **22**, 233 (Jun, 2009).  24. D. J. Kimbrough, M. A. *et al.*, *Neurol Neuroimmunol Neuroinflamm* **1**, e4 (Jun, 2014).  25. F. Di Pauli *et al.*, *Neurol Neuroimmunol Neuroinflamm* **2**, e175 (Dec, 2015).  26. P. Kortvelyessy *et al.*, *Neurol Neuroimmunol Neuroinflamm* **4**, e335 (May, 2017).  27. R. A. Sobel, *Am J Pathol* **170**, 436 (Feb, 2007).  28. N. P. Young *et al.*, *Brain* **133**, 333 (Feb, 2010).  29. C. F. Lucchinetti *et al.*, *Brain* **125**, 1450 (Jul, 2002).  30. E. C. Breij *et al.*, *Ann Neurol* **63**, 16 (Jan, 2008).  31. C. Lucchinetti *et al.*, *Ann Neurol* **47**, 707 (Jun, 2000).  32. A. T. Toosy, D. F. Mason, D. H. Miller, *Lancet Neurol* **13**, 83 (Jan, 2014). |
| --- | --- |
